# Mortality of septic knee arthritis in Korea: risk factors analysis of a large national database

**DOI:** 10.1038/s41598-022-18420-4

**Published:** 2022-08-17

**Authors:** Ho-Jun Choi, Han-Kook Yoon, Hyun-Cheol Oh, Jung-Hwa Hong, Taeyun Choi, Sang-Hoon Park

**Affiliations:** 1 Department of Orthopedic Surgery, Gwangmyeong-Sungae Hospital, Gwangmyeong-si, Republic of Korea; 2 Department of Orthopaedic Surgery, National Health Insurance Service Ilsan Hospital, 100 Ilsan-ro, Ilsandong-gu, Goyang, 10444 Republic of Korea; 3 Research Institute, National Health Insurance Service Ilsan Hospital, Goyang, Republic of Korea

**Keywords:** Diseases, Medical research, Risk factors

## Abstract

This study aimed to analyze the risk factors for mortality of septic knee arthritis in Korea through a large nationwide data research. The National Health Insurance Service-Health Screening database was used to analyze 89,120 hospitalizations for septic knee arthritis between 2005 and 2018. In-hospital, thirty-day, and ninety-day mortality, and their association with patient’s demographic factors, various comorbidities (i.e., cerebrovascular disease, congestive heart failure, and myocardial infarction) and Charlson Comorbidity Index (CCI) were assessed. Secondary outcomes of complications (osteomyelitis, knee arthroplasty, recurrence) were analyzed. The number of hospitalization with septic knee arthritis increased from 1847 cases in 2005 to 8749 cases in 2018. There was no significant difference in mortality after diagnosis of septic knee arthritis between years. The risk of mortality in patients who hospitalized with septic knee arthritis increased in comorbidities like Congestive heart failure, dementia, myocardial infarction, chronic kidney disease. Hazard ratio (HR) decreased in patients who have comorbidities such as rheumatoid arthritis, liver disease, rheumatologic disease. HR for mortality in septic knee arthritis increased in patients with CCI more than 1. The risk factors for mortality in all periods were male sex, old age, high CCI, comorbidities such as congestive heart failure, dementia, myocardial infarction, chronic kidney disease. Efforts to reduce mortality should be concentrate more on patients with these risk factors.

## Introduction

Septic arthritis is a joint infection caused by pathogenic inoculation which are mainly bacterial infections^1–3^. Most of septic arthritis occur in one large peripheral joint such as the knee or the hip joint, but multi-articular septic arthritis at the same time or infections that involve small joints can also occur^4,5^. In the case of septic knee arthritis, it is known to increase mortality and systemic morbidity, and is known as an orthopedic emergency. To prevent this, early diagnosis and treatment are recommended, and based on this, the cartilage and function of the knee joint can be preserved^6^. In the case of septic knee arthritis in adults, Staphylococcus aureus is the most common species, and it can be caused by various species such as streptococci, fungi, and mycobacterium^7,8^. Risk factors for septic knee arthritis include old age, diabetes, rheumatoid arthritis, patients with artificial knee arthroplasty, previous intra-articular corticosteroid injection, skin infections, degenerative arthritis, immunocompromised patients, intravenous drug abuse^6,9^. The incidence of septic arthritis was reported to be 2–10 cases per 100,000 and more than 50% of them occur in the knee joint^7,10–12^. When such septic knee arthritis occurs, long-term hospitalization is required for antibiotic treatment and surgical treatment, and complications may occur even after treatment. Because septic knee arthritis is difficult to diagnose and treat, and its morbidity and mortality are high, it is necessary to study the cause of the increase in mortality. However, there are few studies about the risk factor analysis, or large-scale studies. Moreover, the risk of mortality associated with each comorbidity is also unclear^13^. Therefore, we aimed to analyze the risk factors for mortality of septic knee arthritis in Korea through a large nationwide data research.

## Results

**Figure 1:**
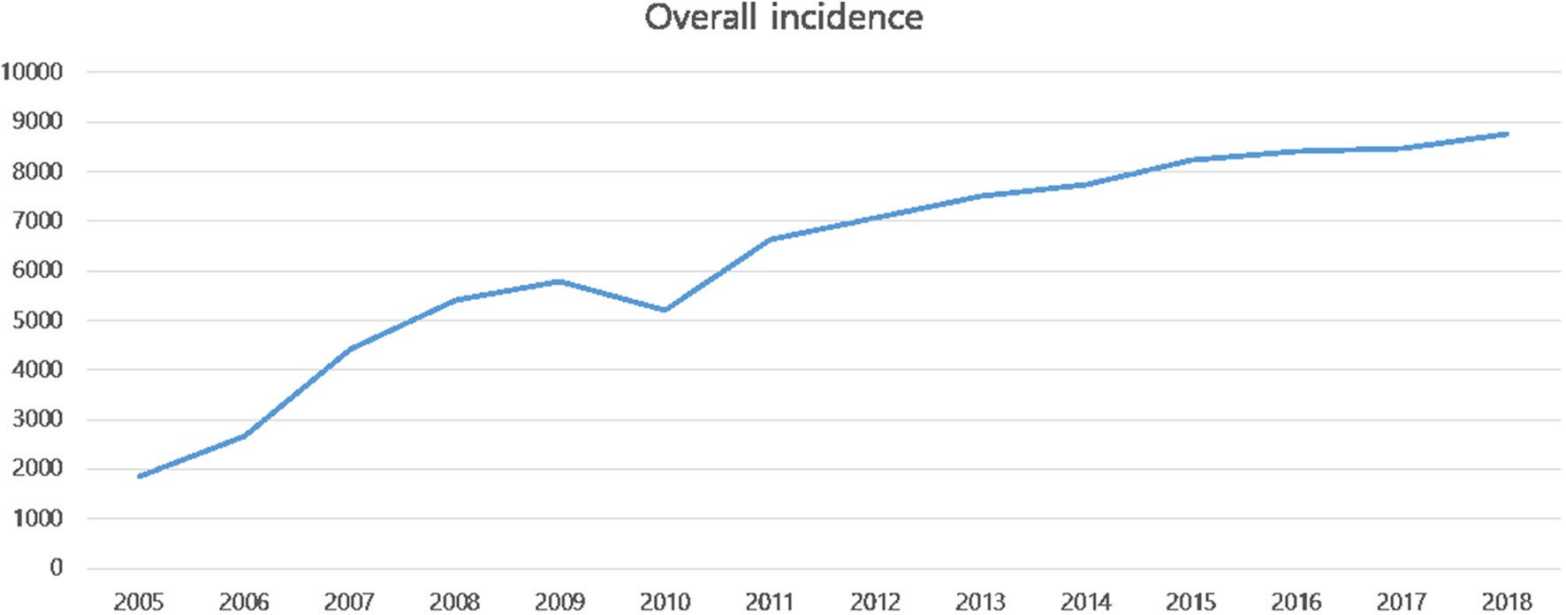
The incidence of septic knee arthritis by year.

**Figure 2:**
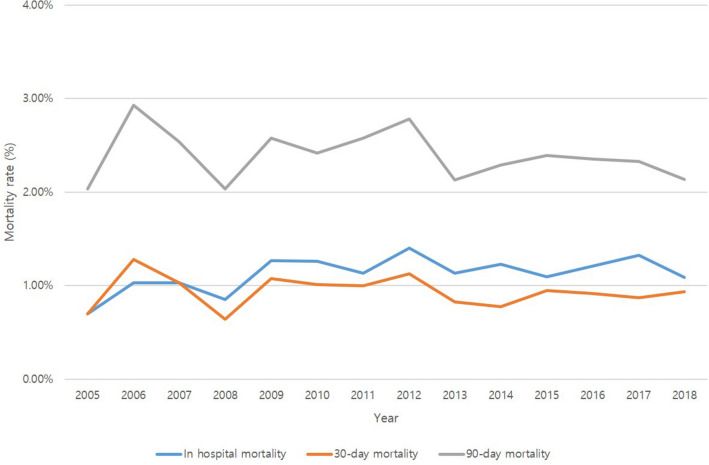
The mortality of septic arthritis by year.

From 2005 to 2018, numbers of hospitalization for septic knee arthritis were 89,120 cases in this study. The number of hospitalization with septic knee arthritis increased from 1847 cases in 2005 to 8749 cases in 2018 (Fig. 1). And incidence per 100,000 of septic arthritis was increased from 4.072 in 2015 to 15.298 in 2018 (Table 1) . The age-specific trend of septic knee arthritis was 2354 cases in their 20 s, 24,119 cases in their 70 s, and showed an increase in the elderly, 13,933 cases in their 80 s and 1634 cases in their 90 s, which decreased after the age of 80. There were 39,912 males (44.93%) and 48,919 females (55.07%), and the number of cases of septic knee arthritis was higher in females (Table 2). After diagnosis of septic arthritis, the hospital stay was longer than 4 weeks in 28% of the total cases, requiring long-term hospitalization (Table 2) . After diagnosis of septic knee arthritis, in-hospital mortality were 1789 cases (2.01%), 953 cases (1.07%) within 30 days, and 2403 cases (2.70%) within 90 days. There was no significant difference in mortality after diagnosis of septic knee arthritis between years (Fig. 2). In secondary outcomes of septic knee arthritis, such as recurrence, HR increased in patients with comorbidities such as gout, rheumatoid arthritis, congestive heart failure, chronic pulmonary disease, dementia, myocardial infarction, and chronic kidney disease. In the case of osteomyelitis, HR was elevated in congestive heart failure, chronic pulmonary disease, hypertension, peripheral vascular disease, and chronic kidney disease. Also, in arthroplasty, HR was elevated in rheumatoid arthritis, chronic pulmonary disease, hypertension, diabetes, peripheral vascular disease, and rheumatologic disease (Table 7).

**Table 1:** Incidence of septic knee arthritis per 100,000.

Year	Septic arthritis patients	Incidence per 100,000
2005	1723	4.072
2006	2353	5.561
2007	3825	9.040
2008	4510	10.658
2009	4718	11.150
2010	5098	12.048
2011	5325	12.585
2012	5670	13.400
2013	5931	14.017
2014	6088	14.388
2015	6366	15.045
2016	6453	15.250
2017	6291	14.868
2018	6473	15.298

**Table 2:** Characteristics of the study participants.

Characteristics	N (%)
**Number of cases**	89,120
**Age**	
20–29	2354 (2.65)
30–39	4797 (5.4)
40–49	8378 (9.43)
50–59	14,670 (16.51)
60–69	18,893 (21.27)
70–79	24,119 (27.15)
80–89	13,933 (15.68)
90–99	1634 (1.84)
≥ 100	53 (0.06)
**Gender**	
Male	39,912 (44.93)
Female	48,919 (55.07)
**Charlson Comorbidity Index Score**	
0	34,184 (38.36)
1	24,880 (27.92)
2	13,978 (15.68)
3 and greater	16,078 (18.04)
**Length of hospital day**	
≤ 7 days	15,752 (17.68)
8–14 day	20,142 (22.6)
15–21 day	17,431 (19.56)
22–28 day	10,843 (12.17)
29–60 day	18,138 (20.35)
≥ 2 months	6814 (7.65)

### Factors affecting in-hospital mortality of septic knee arthritis

The number of in-hospital mortality of septic knee arthritis were 1789 cases. Table 3 shows the results of a series of univariable analyses using the Cox model. Among the patient's demographics, female sex and old age was significantly associated with in-hospital mortality. Hazard ratio increased from 60 years of age or older. Compared with those with CCI of 0, patients with CCI of 1 was not statistically different but patients with CC of 2 (HR = 1.29; 95% CI = 1.09–1.56, p = 0.0082) and ≥ 3 (HR = 2.19; 95% CI = 1.88–2.55, p < 0.0001) was associated with in-hospital mortality. HR increased in patients who underwent surgery (HR = 2.06; 95% CI = 1.85–2.29, p < 0.0001) but antibiotics treatment was not associated with mortality. Rheumatoid arthritis (HR = 1.46; 95% CI = 1.15–1.86, p = 0.0021), congestive heart failure (HR = 1.37; 95% CI = 1.15–1.62, p = 0.0003), dementia (HR = 1.43; 95% CI = 1.24–1.65, p < 0.0001), myocardial infarction (HR = 1.52; 95% CI = 1.24–1.89, p = 0.0002), chronic kidney disease (HR = 1.45; 95% CI = 1.23–1.72, p < 0.0001) were associated with in-hospital mortality. HR decreased in patients who have osteoarthritis (HR = 0.76; 95% CI = 0.65–0.88, p = 0.0004), liver disease (HR = 0.76; 95% CI = 0.66–0.88, p = 0.0002), rheumatologic disease (HR = 0.61; 95% CI = 0.50–0.73, p < 0.0001). Gout, cerebrovascular disease, chronic pulmonary disease, hypertension, diabetes, malignancy, peripheral vascular disease was not statistically significant (Table 6).

**Table 3:** Cox proportional hazard models of in-hospital mortality by variables.

Variables	N	Univariable analysis	
		Hazard ratio (95% CI)	P value
**Gender**			
Male	469	1 (reference)	
Female	729	1.38 (1.25–1.52)	< 0.0001
**Age (years)**			
20–29	6	1 (reference)	
30–39	5	0.42 (0.19–0.90)	0.0267
40–49	17	0.94 (0.52–1.71)	0.8382
50–59	63	1.35 (0.78–2.35)	0.2899
60–69	147	2.27 (1.33–3.89)	0.0028
70–79	379	3.75 (2.21–6.38)	< 0.0001
80–89	472	8.63 (5.08–14.65)	< 0.0001
90–99	105	15.16 (8.74–26.29)	< 0.0001
≥ 100	4	21.29 (8.18–55.41)	< 0.0001
**Charlson Comorbidity Index Score**			
0	276	1 (reference)	
1	264	1.12 (0.98–1.27)	0.0878
2	196	1.29 (1.11–1.49)	0.0007
≥ 3	462	2.35 (2.09–2.65)	< 0.0001
**Surgery**			
No	985	1 (reference)	
Yes	213	2.06 (1.85–2.29)	< 0.0001
**Antibiotics**			
No	276	1 (reference)	
Yes	922	0.96 (0.87–1.07)	0.4742

### Factors affecting thirty-day mortality of septic knee arthritis

After diagnosis of septic knee arthritis, the total number of thirty-day mortality of septic knee arthritis were 953 cases. Table 4 shows the results of a series of univariable analyses using the Cox model. Old age over 60 years were demographic factors that increase the risk of thirty-day mortality of septic knee arthritis. Gender was not associated with thirty-day mortality of septic knee arthritis. HR for thirty-day mortality were 1.26 (95% CI = 1.03 to 1.54, p = 0.0264) and 1.58 (95% CI = 1.27 to 1.97, p < 0.0001) and 3.63 (95% CI = 3.05 to 4.33, p < 0.0001) for patients with Charlson Comorbidity Index (CCI) of 1, 2 and CCI ≥ 3, respectively, compared with patients with CCI of 0. Surgery and antibiotics were not statistically significant. The HR increased in patients with comorbidities such as congestive heart failure (HR = 1.48; 95% CI = 1.20 to 1.83, p = 0.0003), dementia (HR = 1.24; 95% CI = 1.01 to 1.51, p = 0.0359), malignancy (HR = 1.43; 95% CI = 1.14 to 1.80, p = 0.0018), myocardial infarction (HR = 2.05; 95% CI = 1.61 to 2.62, p < 0.0001), chronic kidney disease (HR = 1.62; 95% CI = 1.32 to 1.98, p < 0.0001). But HR decreased in patients with comorbidities such as liver disease (HR = 0.81; 95% CI = 0.67 to 0.97, p = 0.0215), rheumatologic disease (HR = 0.76; 95% CI = 0.61 to 0.94, p = 0.0131) (Table 6).

**Table 4:** Cox proportional hazard models of thirty-day mortality by variables.

Variables	N	Univariable analysis	
		Hazard ratio (95% CI)	P value
**Gender**			
Male	437	1 (reference)	
Female	516	0.96 (0.84–1.09)	0.5166
**Age (years)**			
20–29	3	1 (reference)	
30–39	6	0.761 (0.20 -2.92)	0.762
40–49	17	1.38 (0.44–4.36)	0.5841
50–59	66	2.84 (0.96–8.35)	0.0585
60–69	127	4.39 (1.51–12.74)	0.0065
70–79	311	7.82 (2.72–22.50)	0.0001
80–89	355	16.08 (5.59–46.23)	< 0.0001
90–99	67	25.45 (8.61–75.18)	< 0.0001
≥ 100	1	20.23 (2.97–137.67)	0.0021
**Charlson Comorbidity Index Score**			
0	220	1 (reference)	
1	209	1.26 (1.03–1.54)	0.0264
2	149	1.58 (1.27–1.97)	< 0.0001
≥ 3	375	3.63 (3.05–4.33)	< 0.0001
**Surgery**			
No	822	1 (reference)	
Yes	131	1.02 (0.83–1.25)	0.8603
**Antibiotics**			
No	243	1 (reference)	
Yes	710	1.08 (0.93–1.25)	0.318

### Factors affecting ninety-day mortality of septic knee arthritis

The total numbers of ninety-day mortality of septic knee arthritis were 2403 cases. Table 5 shows the results of a series of univariable analyses using the Cox model. Among the patient's demographics, old age (over 50 years) was significantly associated with ninety-day mortality of septic knee arthritis but gender was not associated. HR for ninety-day mortality were 1.32 (95% CI = 1.16 to 1.50, p < 0.0001) and 1.89 (95% CI = 1.65 to 2.16, p < 0.0001) and 3.81 (95% CI = 3.41 to 4.26, p < 0.0001) for patients with Charlson Comorbidity Index (CCI) of 1, 2 and CCI ≥ 3, respectively, compared with patients with CCI of 0. Surgery was not associated with ninety-day mortality but HR increased in patients who underwent antibiotics treatment (HR = 1.22; 95% CI = 1.11 to 1.34, p < 0.0001). HR increased in patients with comorbidities such as congestive heart failure (HR = 1.37; 95% CI = 1.19 to 1.57, p < 0.0001), dementia (HR = 1.23; 95% CI = 1.09 to 1.39, p = 0.001), malignancy (HR = 1.55; 95% CI = 1.35 to 1.78, p < 0.0001), myocardial infarction (HR = 1.62; 95% CI = 1.36 to 1.91, p < 0.0001), chronic kidney disease (HR = 1.40; 95% CI = 1.23 to 1.60, p < 0.0001). HR decreased in patients who have comorbidities such as rheumatologic arthritis (HR = 0.50; 95% CI = 0.33 to 0.76, p = 0.0011), osteoarthritis (HR = 0.52; 95% CI = 0.37 to 0.73, p = 0.0002), diabetes (HR = 0.87; 95% CI = 0.78 to 0.98, p = 0.0239), liver disease (HR = 0.77; 95% CI = 0.68 to 0.86, p < 0.0001), rheumatologic disease (HR = 0.76; 95% CI = 0.66 to 0.87, p < 0.0001) (Table 6).

**Table 5:** Cox proportional hazard models of ninety-day mortality by variables.

Variables	N	Univariable analysis	
		Hazard ratio (95% CI)	p-value
**Gender**			
Male	1059	1 (reference)	
Female	1343	1.03 (0.94–1.12)	0.57
**Age (years)**			
20–29	6	1 (reference)	
30–39	13	0.93 (0.36–2.41)	0.8853
40–49	43	1.81 (0.79–4.13)	0.1611
50–59	129	3.05 (1.38–6.72)	0.0057
60–69	312	5.74 (2.64–12.47)	< 0.0001
70–79	811	11.31 (5.22–24.49)	< 0.0001
80–89	893	22.13 (10.22–47.92)	< 0.0001
90–99	187	41.72 (19.03–91.45)	< 0.0001
≥ 100	8	64.66 (23.26–179.74)	< 0.0001
**Charlson Comorbidity Index Score**			
0	532	1 (reference)	
1	522	1.32 (1.16–1.50)	< 0.0001
2	413	1.89 (1.65–2.16)	< 0.0001
≥ 3	936	3.81 (3.41–4.26)	< 0.0001
**Surgery**			
No	2052	1 (reference)	
Yes	351	1.13 (0.99–1.27)	0.0612
**Antibiotics**			
No	561	1 (reference)	
Yes	1842	1.22 (1.11–1.34)	< 0.0001

**Table 6:** Cox proportional hazard models of in-hospital, thirty-day, ninety-day mortality by variables.

Variables	Multivariable analysis					
	In-hospital mortality		Thirty-day mortality		Ninety-day mortality	
	Hazard ratio (95% CI)	P value	Hazard ratio (95% CI)	P value	Hazard ratio (95% CI)	P value
**Gout**						
No	1 (reference)		1 (reference)		1 (reference)	
Yes	0.87 (0.74–1.02)	0.0897	0.87 (0.70–1.07)	0.1775	0.93 (0.81–1.06)	0.2529
**Rheumatoid arthritis**						
No	1 (reference)		1 (reference)		1 (reference)	
Yes	1.46 (1.15–1.86)	0.0021	0.42 (0.23–0.76)	0.0046	0.52 (0.37–0.73)	0.0002
**Osteoarthritis**						
No	1 (reference)		1 (reference)		1 (reference)	
Yes	0.76 (0.65–0.88)	0.0004	0.90 (0.74–1.09)	0.2827	0.88 (0.78–0.99)	0.0484
**Cerebrovascular disease**						
No	1 (reference)		1 (reference)		1 (reference)	
Yes	1.14 (0.98–1.32)	0.0901	1.18 (0.98–1.42)	0.0844	1.12 (0.99–1.26)	0.0548
**Congestive heart failure**						
No	1 (reference)		1 (reference)		1 (reference)	
Yes	1.37 (1.15–1.62)	0.0003	1.48 (1.20–1.83)	0.0003	1.37 (1.19–1.57)	< .0001
**Chronic pulmonary disease**						
No	1 (reference)		1 (reference)		1 (reference)	
Yes	0.87 (0.75–1.01)	0.0754	0.94 (0.77–1.15)	0.542	1.08 (0.96–1.21)	0.2003
**Hypertension**						
No	1 (reference)		1 (reference)		1 (reference)	
Yes	0.90 (0.76–1.08)	0.2539	0.94 (0.75–1.17)	0.5644	0.97 (0.84–1.12)	0.678
**Dementia**						
No	1 (reference)		1 (reference)		1 (reference)	
Yes	1.43 (1.24–1.65)	< .0001	1.24 (1.01–1.51)	0.0359	1.23 (1.09–1.39)	0.001
**Diabetes**						
No	1 (reference)		1 (reference)		1 (reference)	
Yes	0.93 (0.80–1.07)	0.2914	0.94 (0.78–1.13)	0.4768	0.87 (0.78–0.98)	0.0239
**Liver disease**						
No	1 (reference)		1 (reference)		1 (reference)	
Yes	0.76 (0.66–0.88)	0.0002	0.81 (0.67–0.97)	0.0215	0.77 (0.68–0.86)	< .0001
**Malignancy**						
No	1 (reference)		1 (reference)		1 (reference)	
Yes	1.20 (0.99–1.46)	0.0644	1.43 (1.14–1.80)	0.0018	1.55 (1.35–1.78)	< .0001
**Myocardial infarction**						
No	1 (reference)		1 (reference)		1 (reference)	
Yes	1.52 (1.24–1.89)	0.0002	2.05 (1.61–2.62)	< .0001	1.62 (1.36–1.91)	< .0001
**Peripheral vascular disease**						
No	1 (reference)		1 (reference)		1 (reference)	
Yes	1.04 (0.88–1.24)	0.6274	1.13 (0.90–1.40)	0.2894	1.11 (0.97–1.28)	0.1331
**Rheumatologic disease**						
No	1 (reference)		1 (reference)		1 (reference)	
Yes	0.61 (0.50–0.73)	< .0001	0.76 (0.61–0.94)	0.0131	0.76 (0.66–0.87)	< .0001
**Chronic kidney disease**						
No	1 (reference)		1 (reference)		1 (reference)	
Yes	1.45 (1.23–1.72)	< .0001	1.62 (1.32–1.98)	< .0001	1.4 (1.23–1.60)	< .0001

## Discussion

The National Health Insurance Service (National Health Insurance Service-HealthScreening; NHIS-HealS) based big data has great significance as a large-scale cohort study. This study is a large-scale big data study for about 15 years from 2005 to 2018, and through this, the overall prevalence and trend of septic knee arthritis in South Korea can be evaluated. It was possible to analyze the difference in the risk and mortality of each disease according to gender, age, and comorbidities. For each underlying disease, it is possible to determine the risk of complications that may occur after diagnosis of septic knee arthritis (Table 7).

**Table 7:** Cox proportional hazard models of secondary outcomes.

Variables	Multivariable analysis					
	Osteomyelitis		Arthroplasty		Recurrence	
	Hazard ratio (95% CI)	P value	Hazard ratio (95% CI)	P value	Hazard ratio (95% CI)	P value
**Gout**						
No	1 (reference)		1 (reference)		1 (reference)	
Yes	0.60 (0.53–0.67)	< .0001	0.75 (0.70–0.80)	< .0001	1.16 (1.10–1.22)	< .0001
**Rheumatoid arthritis**						
No	1 (reference)		1 (reference)		1 (reference)	
Yes	0.96 (0.79–1.17)	0.7008	1.21 (1.10–1.34)	0.0001	1.31 (1.18–1.45)	< .0001
**Osteoarthritis**						
No	1 (reference)		1 (reference)		1 (reference)	
Yes	0.96 (0.88–1.04)	0.3212	0.66 (0.63–0.7)	< .0001	0.87 (0.83–0.91)	< .0001
**Cerebrovascular disease**						
No	1 (reference)		1 (reference)		1 (reference)	
Yes	1.01 (0.90–1.12)	0.9043	0.89 (0.84–0.95)	0.0002	0.99 (0.92–1.05)	0.69
**Congestive heart failure**						
No	1 (reference)		1 (reference)		1 (reference)	
Yes	1.21 (1.05–1.38)	0.0064	1.06 (0.99–1.15)	0.1068	1.20 (1.11–1.30)	< .0001
**Chronic pulmonary disease**						
No	1 (reference)		1 (reference)		1 (reference)	
Yes	1.29 (1.18–1.41)	< .0001	1.09 (1.04–1.15)	0.0011	1.09 (1.03–1.15)	0.03
**Hypertension**						
No	1 (reference)		1 (reference)		1 (reference)	
Yes	1.14 (1.03–1.25)	0.0123	1.14 (0.18–1.20)	< .0001	0.91 (0.85–0.97)	0.0022
**Dementia**						
No	1 (reference)		1 (reference)		1 (reference)	
Yes	1.0 (0.87–1.15)	0.99	0.79 (0.73–0.86)	< .0001	1.47 (1.37–1.58)	< .0001
**Diabetes**						
No	1 (reference)		1 (reference)		1 (reference)	
Yes	1.06 (0.97–1.16)	0.212	1.09 (1.03–1.14)	0.0017	0.96 (0.90–1.01)	0.1247
**Liver disease**						
No	1 (reference)		1 (reference)		1 (reference)	
Yes	0.96 (0.88–1.04)	0.3232	1.04 (0.99–1.09)	0.1388	0.87 (0.83–0.92)	< .0001
**Malignancy**						
No	1 (reference)		1 (reference)		1 (reference)	
Yes	0.87 (0.74–1.01)	0.0734	0.84 (0.76–0.92)	0.0003	1.01 (0.92–1.11)	0.8007
**Myocardial infarction**						
No	1 (reference)		1 (reference)		1 (reference)	
Yes	1.06 (0.87–1.29)	0.5602	0.86 (0.77–0.97)	0.0158	1.17 (1.04–1.31)	0.0083
**Peripheral vascular disease**						
No	1 (reference)		1 (reference)		1 (reference)	
Yes	1.20 (1.07–1.34)	0.002	1.14 (1.07–1.21)	< .0001	1.07 (0.99–1.15)	0.0707
**Rheumatologic disease**						
No	1 (reference)		1 (reference)		1 (reference)	
Yes	1.07 (0.97–1.17)	0.1863	1.23 (1.16–1.29)	< .0001	1.06 (0.99–1.12)	0.0534
**Chronic kidney disease**						
No	1 (reference)		1 (reference)		1 (reference)	
Yes	1.37 (1.21–1.56)	< .0001	0.87 (0.80–0.95)	0.0026	1.51 (1.40–1.64)	< .0001

Although septic knee arthritis is not a common disease, it is one of the diseases that require emergency surgery or treatment^6^. The incidence of septic arthritis is about 4–10 per 100,000 patient-years per year^6,7,10,11^. Two recent nationwide studies from the United Kingdom and New Zealand reported that the incidence of septic arthritis is 6.7 and 12.2 per 100,000^14,15^. Two studies reported that the incidence of septic arthritis is increasing^14,15^. In our study, the number of cases diagnosed with septic knee arthritis increased from 1847 cases in 2005–8749 cases in 2018. Although this increase in incidence can be explained by increased reporting due to advances in diagnostic methods and advances in the medical system, it is also possible that the rate of septic arthritis has actually increased. Intra-articular infections are a rare but well-known complication of joint injections and surgery. An increase in procedures such as joint injection may be thought to be related to an increase in iatrogenic joint infection. In terms of gender, there were 39,912 male cases (44.93%) and 48, 919 female cases (55.07%), which were more common in females, and the severity and risk of the disease were also higher in female patients. In this study, HR for in-hospital mortality in female compared to male were 1.38 (95% CI = 1.25 to 1.52, p < 0.0001), respectively. Gender factor was also found to affect the severity and risk of septic knee arthritis. The other study reported that male patients had a 33% increased death risk^16^.

It is known as a disease that can lead to death if appropriate emergency treatment is not provided. In other studies, the mortality rate is reported to be 4–15%, the incidence of osteomyelitis is about 8%, and poor clinical results are reported in more than 20–30%^6,7,12,17,18^. But, the other nationwide study in Iceland reported lower mortality such as 2.7%^11^. In this study, between 2005 and 2018, in-hospital mortality was 2.01%, thirty-day mortality was 1.07%, and 90-day mortality was 2.70%. And there was no significant difference in mortality between 2005 and 2018.

In this study, the risk factors for mortality in all periods were old age, high CCI, comorbidities such as congestive heart failure, dementia, myocardial infarction, chronic kidney disease. And HR decreased in patients with liver disease and rheumatologic disease. The other studies also reported that, in terms of patient demographics, male sex and old age were reported as a risk factor for mortality after diagnosis of septic arthritis^16,17,19–21^. As in this study, other institutions reported that coronary artery disease, cerebrovascular disease, chronic kidney disease, malignancy among comorbidities were risk factors for mortality after diagnosis of septic arthritis^16,17,20^. In the case of elderly patients with these comorbidities, after diagnosis of septic knee arthritis, the mortality increases due to systemic infection and exacerbation of the inflammatory response. Diabetes was not statistically significant as a risk factor for mortality in this study, but in most other studies, it was reported as a risk factor for mortality^16,17,19–21^.

In other studies, rheumatoid arthritis and liver disease were reported as risk factors for increasing mortality after diagnosis of septic arthritis, but in this study, liver disease, rheumatologic disease, and rheumatologic arthritis were associated with decreased HR for mortality^16,17,20,21^. Rheumatoid arthritis is a known risk factor for septic arthritis^6,22,23^. Although rheumatoid arthritis can be considered as risk factors for septic knee arthritis, it can be evaluated as better than idiopathic septic knee arthritis in terms of prognosis of septic knee arthritis. Rheumatoid arthritis is difficult to differentiate from septic arthritis. So it was treated as septic arthritis without being confirmed in culture and it is assumed that people who have those comorbidities were associated with decreased HR for mortality in this study.

There are some limitations to this study. First, the limitation of this study is that the study period (2005–2018) is relatively short and there are characteristics of the disease that require long-term follow-up, so it has the disadvantage that it does not reflect the overall natural history of the disease. Comparing the data from the 1990s and the 2000s, which achieved the development of drugs and treatments, would have been useful in analyzing whether these treatment policies and drugs were effective in preventing the aggravation of diseases. Before 2005, there was no data in the NHIS database, so there was a limit to the investigation for long term study period^13^. Second, since it is data based on the health insurance service billing data code, it is believed that there are many codes and data that are actually missing in the claim process of each hospital. In addition, there is a limitation that other factors that may affect the patient's knee joint function and condition, such as smoking, drinking alcohol, lifestyle, mental health, and the patient's condition before surgery, could not be observed or analyzed. Third, in most cases, antibiotic treatment is required for the treatment of septic arthritis. But in this report, it was investigated that some septic knee arthritis patients received no antibiotic treatment. This result is because some cases in which antibiotic treatment given to patients with septic arthritis were not covered by national health insurance service system. Due to the nature of big data research, it is impossible to determine the severity of each disease and the underlying disease can only be identified by whether or not the disease is listed in the code. Unlike other studies, diabetes mellitus was not associated with mortality. Additional study is needed in future.

## Conclusion

We studied the trend and specific comorbidities associated with mortality of septic knee arthritis. Demographic factors for mortality of septic knee arthritis were old age. Patients with comorbidities such as congestive heart failure, dementia, myocardial infarction, and chronic kidney disease showed increased HR after diagnosis of septic knee arthritis. In the case of patients with these comorbidities, treatment should always be carried out with the possibility of exacerbation of other related complications, and management and monitoring of the patient's general condition should be essential.

## Materials and methods

This study is retrospective cohort study using customized data provided by the National Health Insurance Service (National Health Insurance Service-HealthScreening; NHIS-HealS)^13^. This study used NHIS-NSC data made by National Health Insurance Service (NHIS). The Institutional Review Board of National Health Insurance Service Ilsan Hospital (NHIMC 2021-11-012) approved this retrospective Health Insurance Portability and Accountability Act-compliant cohort study and waived the informed consent from the participants, because this study was expected to present no or minimal risk of harm to the participants, and all the data used were anonymized^13^. All methods were performed in accordance with relevant guidelines and regulations^13^. The authors alone are responsible for the content and writing of the paper. In Korea there is an obligatory National Health Insurance system with universal coverage^13^. NHIS-HealS database has reimbursement records from all medical institutions in Korea^13^. Septic knee arthritis was investigated by their diagnostic codes in the NHIS-HealS database. Among patients diagnosed with septic knee arthritis (diagnostic codes : M0096), patients above 20 years of age who had been charged for hospitalization between January 1, 2005 and December 31, 2018 were included in the study^13^. In Korea, the diagnosis code M00.96 is often selected after confirming the knee joint fluid analysis before the bacterial culture result is confirmed, and other codes (M00.06, M00.16, M00.26, M00.86) are excluded. Patients who underwent total knee arthroplasty were excluded. Patients hospitalized for septic knee arthritis between 2002 and 2004 were also excluded. A total of 89,120 hospitalizations were included.

In this case, we investigated all-cause mortality and cases of mortality were divided into groups according to the period from the day of hospitalization to the day of death (in-hospital mortality, thirty-day mortality, ninety-day mortality). Definition of ‘in-hospital mortality’ is the mortality cases within in-hospital period and definition of ‘thirty-day mortality’ is the mortality cases within 30 days after discharge, and definition of ‘ninety-year mortality’ means death between the day of discharge to 90 days. This was to confirm the difference between the period of death after hospitalization and to determine whether the effect of comorbidity differs by the period from hospitalization to mortality^13^. Patient demographic factors including age and sex was investigated and comorbidities was collected using International Classification of Diseases 10 diagnostic codes reported in NHIS^24^. We calculated the Charlson Comorbidity Index (CCI) for each patient and divided CCI into four categories : 0, 1, 2 and ≥ 3, as originally proposed by Charlson et al^25^. The comorbidities included for the analysis were gout, rheumatoid arthritis, osteoarthritis, cerebrovascular disease, congestive heart failure, chronic pulmonary disease, hypertension, dementia, diabetes, liver disease, malignancy, myocardial infarction, peripheral vascular disease, rheumatologic disease, chronic kidney disease.

For all analyses, SAS Enterprise 7.1 (SAS Inc., Cary, NC, USA) was used. We performed conditional Cox proportional hazards model analyses of the comorbidities. Hazard ratios (HRs) and 95% confidence intervals (CIs) are presented. The level of significance was maintained at a *P* value < 0.05.
